# Supplementary material for: VGLUT2-expressing neurons in the vestibular nuclear complex mediate gravitational stress-induced hypothermia in mice
Source: Commun Biol. 2020 May 8;3:227. doi: 10.1038/s42003-020-0950-0 (PMC7210111; doi:10.1038/s42003-020-0950-0)
Supplement: Supplementary file 1 — Supplementary Information [file 42003_2020_950_MOESM1_ESM.pdf]

Supplementary Fig. 1

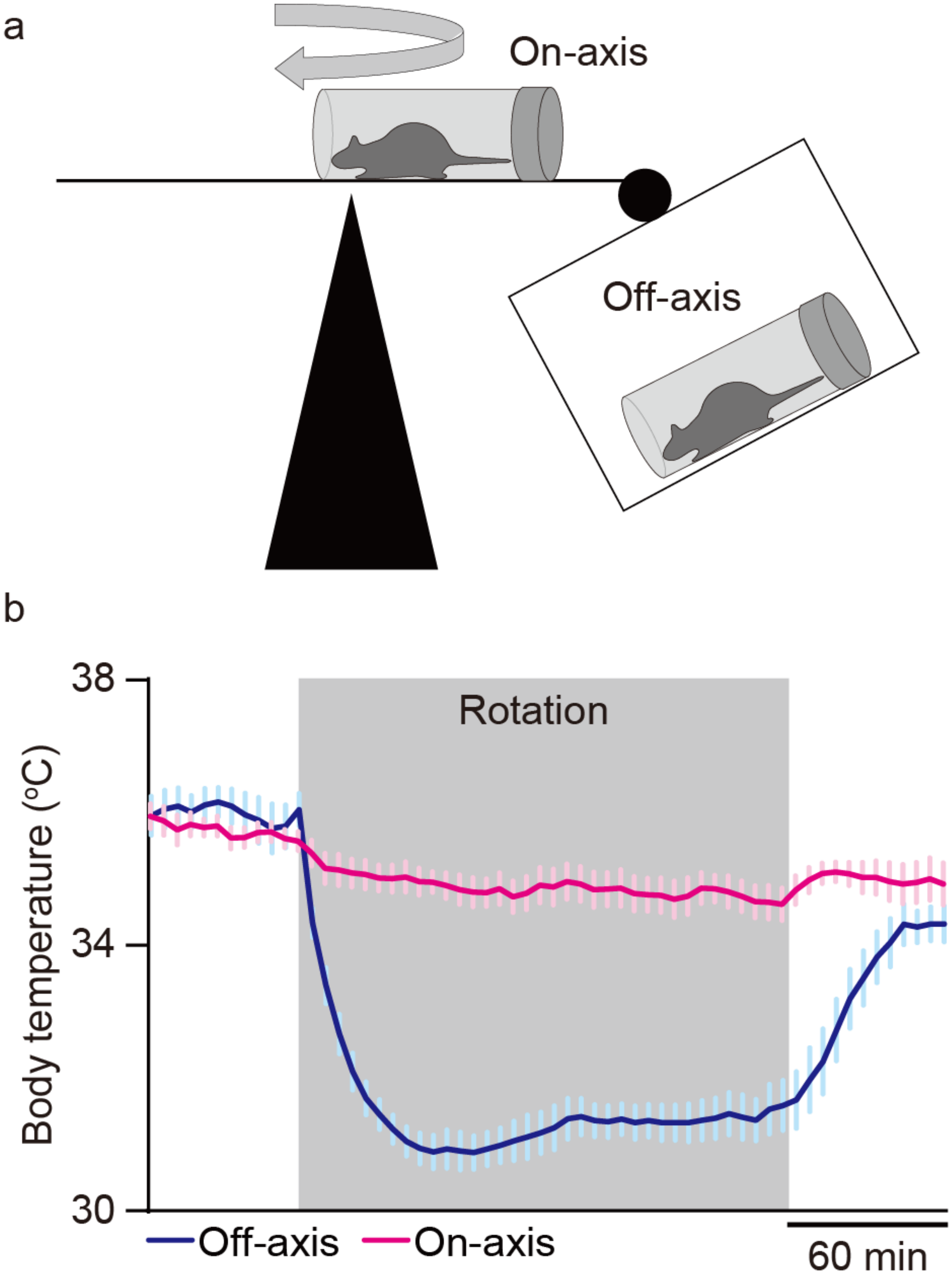

Supplementary Fig. 1: Changes in body temperature (BT) during on-axis rotation

(**a**) A schema of on-axis and off-axis rotations. The rpm of the on-axis rotation that created the environment was the same as that of the off-axis rotation. (**b**) Averaged time-dependent changes in the BT in mice with exposure to on-axis or off-axis rotation. The rotation was applied for 3 h (gray background). Data are means ± SEM (error bars).

Supplementary Fig. 2

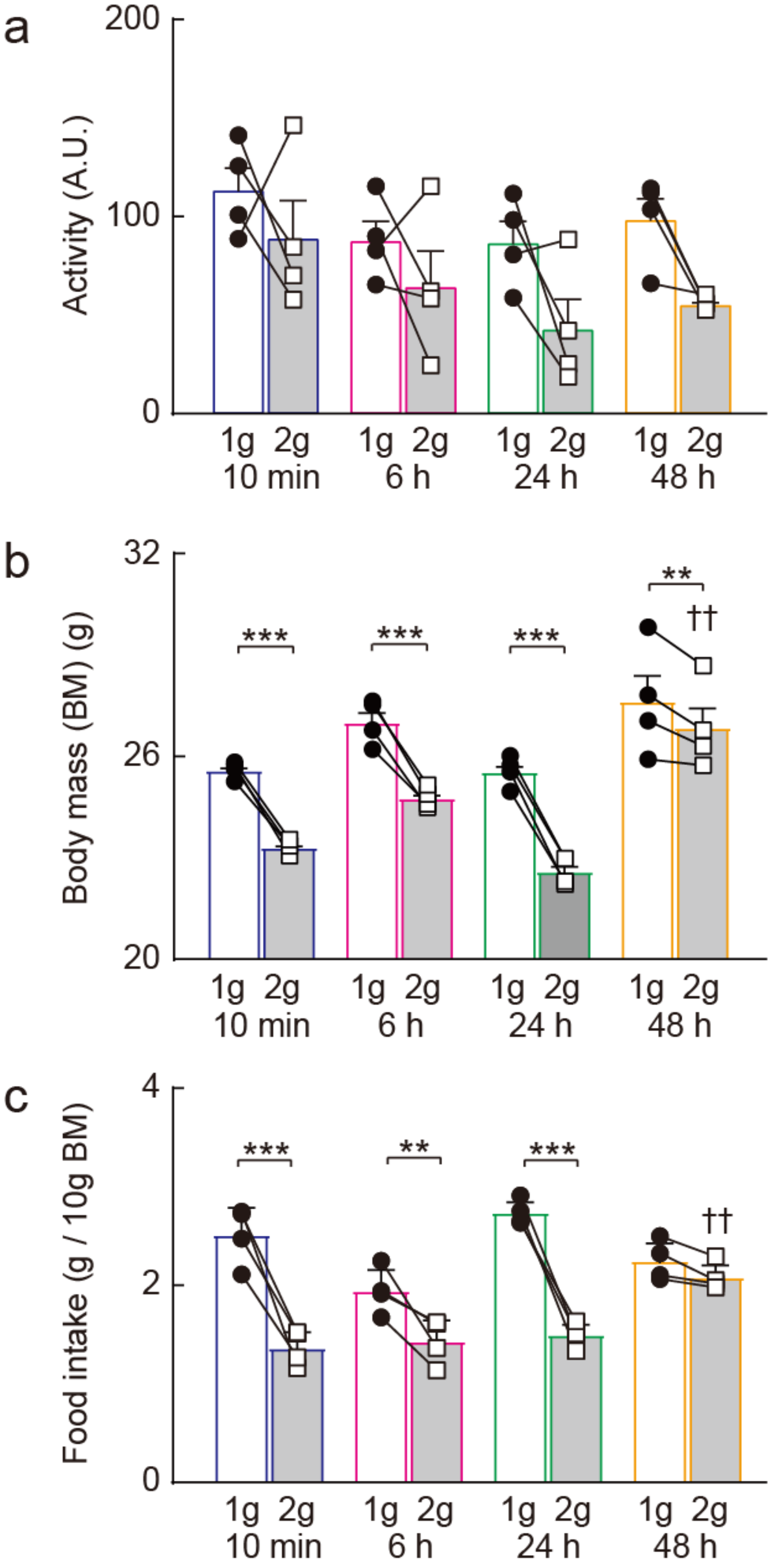

Supplementary Fig. 2: The effect of the slope of gravitational change on the activity, body mass and food intake.

(**a**) Summarized data of activity during exposure to the 2g environment. Each value in the 1g column was obtained by averaging the values of the 24 h prior to the start of the increase in gravity, while each value in the 2g column represented the averaged values during exposure to hypergravity for 48 h. Two-way ANOVA; F (3,12) = 0.2762 (Interaction), $P = 0.8415$. (**b**) Summarized data of body mass (BM) prior to and following the hypergravity load. Each value in the 1g and 2g columns was measured before and after hypergravity loads, respectively. Two-way ANOVA with the Bonferroni's multiple comparisons tests; F (3,12) = 22.43, $P < 0.0001$ (Interaction) (**c**) Summarized data of food intake prior to and during the hypergravity loads. Each value in the 1g and 2g columns indicates the amount of food intake for the 48 h before and during hypergravity loads, respectively. The values were normalized by body mass. Two-way ANOVA with the Bonferroni's multiple comparisons tests; F (3,12) = 18.23, $P < 0.0001$ (Interaction). For all statistical analyses, **$P < 0.01$ and ***$P < 0.001$ 1g vs. 2g; ††$P < 0.01$ vs. 10 min, 6 h and 24 h in 2g. Detailed information on the statistical analyses is reported in Supplementary Table 2.

Supplementary Fig. 3

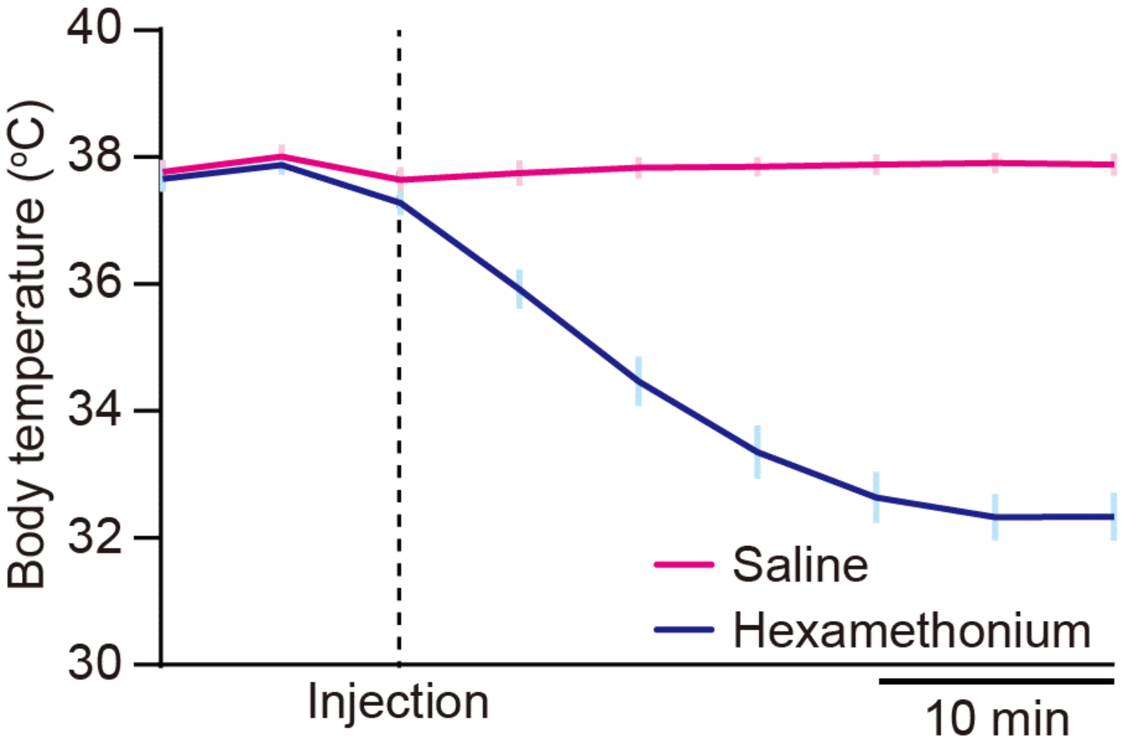

Supplementary Fig. 3

Changes in body temperature (BT) following hexamethonium administration

Averaged time-dependent changes in mice BT following the administration of either hexamethonium or saline at room temperature. The vertical dotted line represents the time of either hexamethonium or saline administration. Data are means ± SEM (error bars).

Supplementary Fig. 4

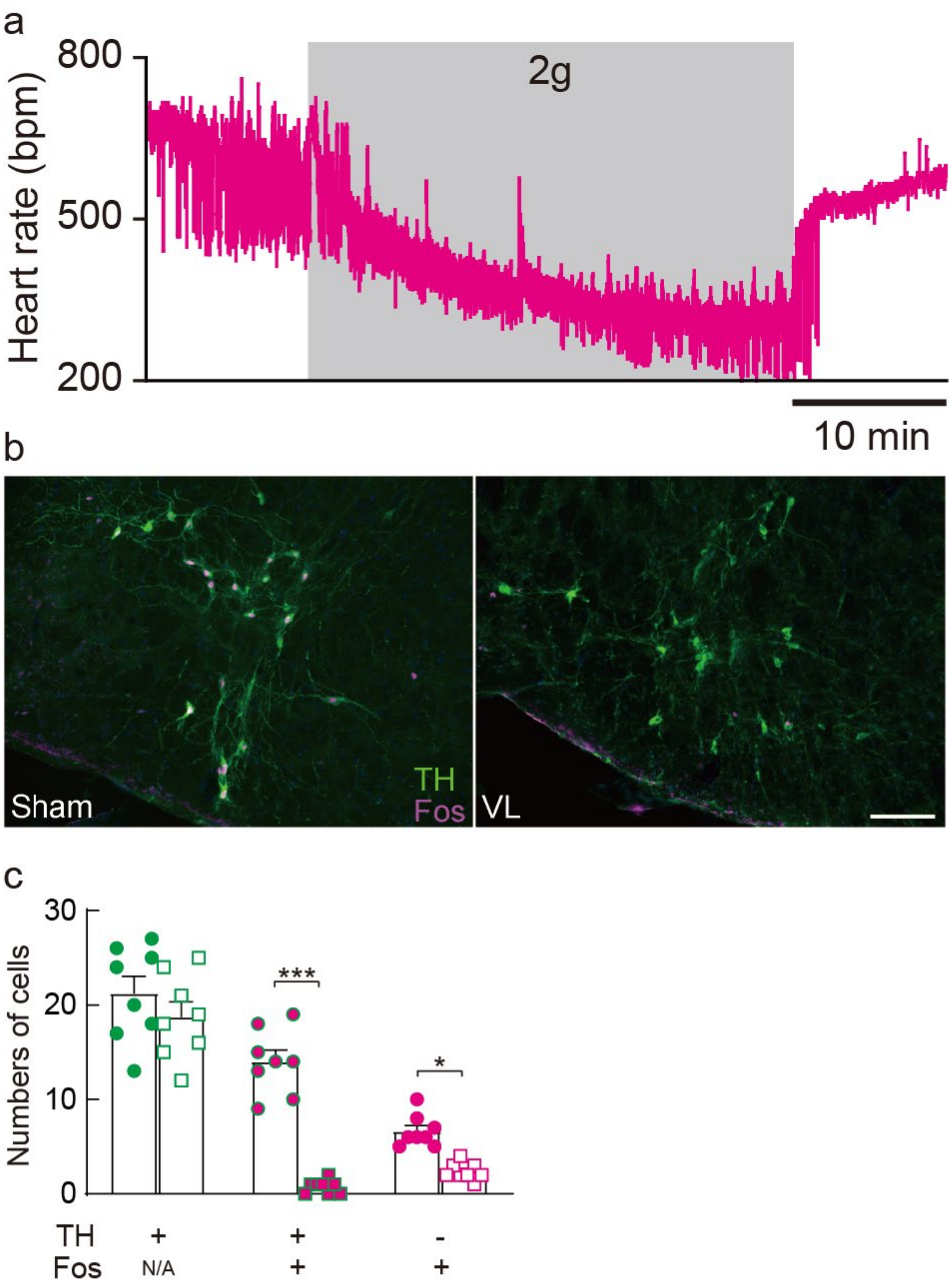

Supplementary Fig. 4: Activation of C1 neurons induced by vestibular stimulation
(**a**) Representative data of the time-dependent changes in the heart rates (HR) of mice during the 2g load. Exposure to the 2g environment lasted for 30 min. (**b**) The 2g load was applied for 90 min to examine the c-fos expression in C1 neurons (TH-expressing neurons in the rostral ventrolateral medulla). Mice underwent a vestibular lesion (VL) or its sham operation (Sham). Scale bar, 100 μm. (**c**) Summarized data of c-fos expression in C1 neurons induced by 2g load for 90 min using Sham and VL mice. Two-way ANOVA with Bonferroni's post-hoc test; $F (2, 42) = 13.05$ $P < 0.0001$ (Interaction). Single or triple significant symbols indicate $P < 0.05$, or $P < 0.001$, respectively. Detailed information on the statistical analyses is reported in Supplementary Table 2.

Supplementary Fig. 5

a

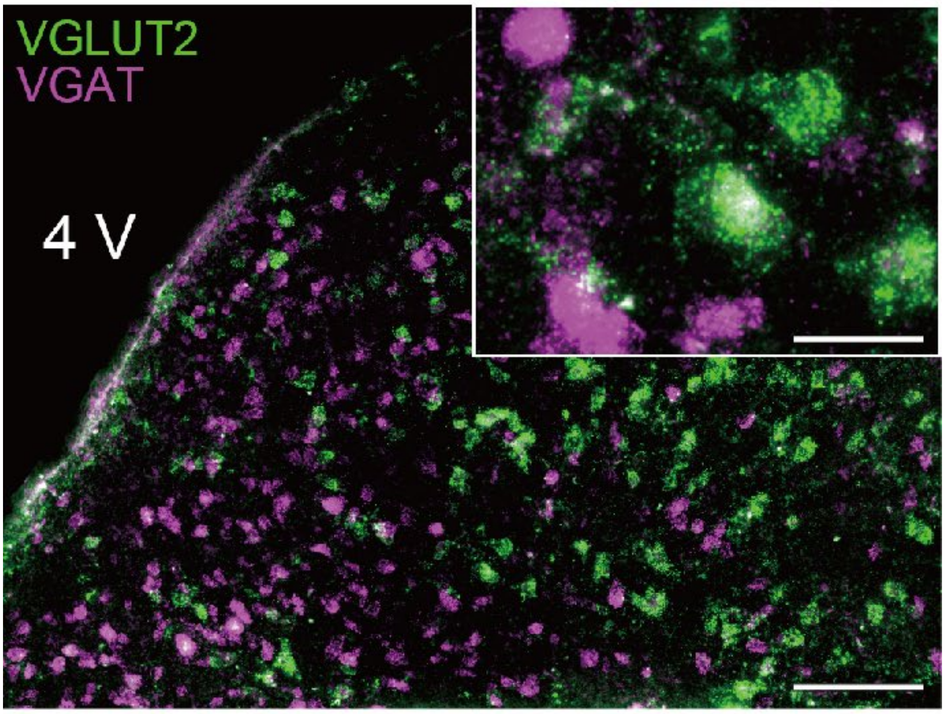

b

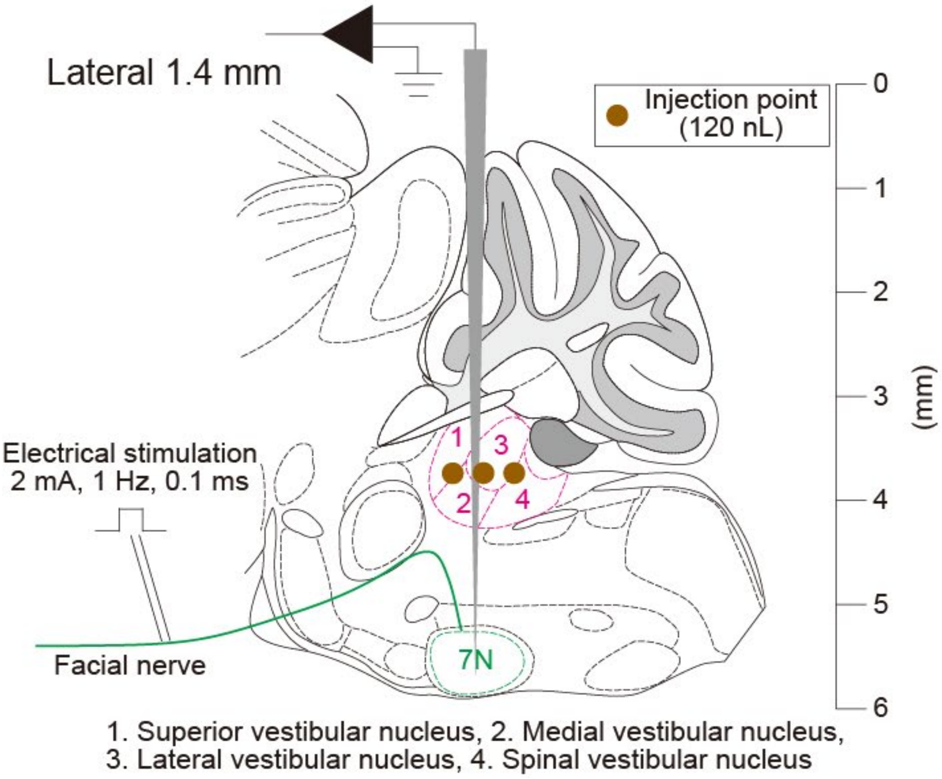

Supplementary Fig. 5: Injection of the viral vector into the vestibular nuclear complex (VNC)

(**a**) Gene expression of the VGLUT2 (green) and VGAT (magenta) in the VNC. The label 4 V represents the 4th ventricle. The scale bar is 100 (main) and 50 (inset) μm. (**b**) Injection of viral vector to express either ChR2 or eArch in VGLUT2- and VGAT-expressing neurons in the VNC. The facial nucleus, which is found through evoked potential induced by electrical stimulation of the facial nerve, is regarded as a landmark to find the VNC.

Supplementary Fig. 6

a

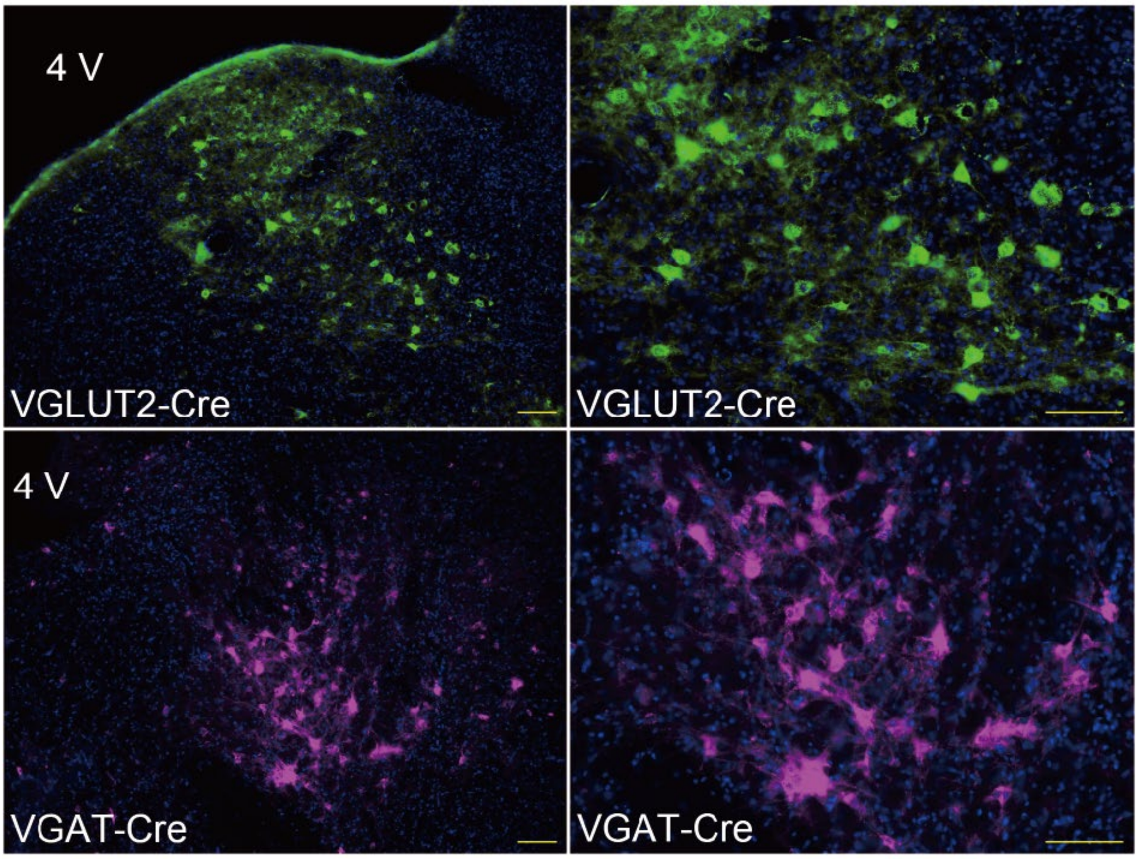

b

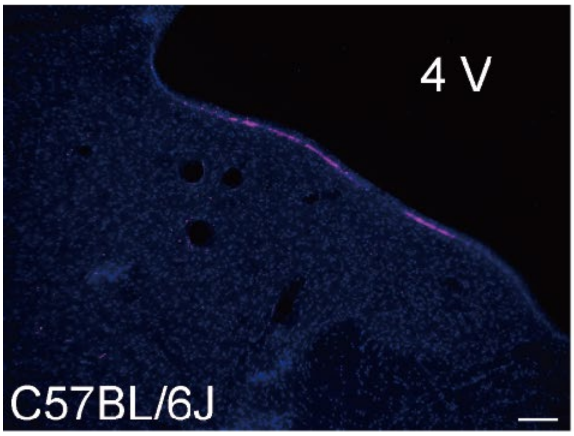

Supplementary Fig. 6: Expression of hm3D(Gq) in VGLUT2- and VGAT-expressing neurons in the vestibular nuclear complex (VNC)

Representative images of hm3D(Gq) expression in VGLUT2- and VGAT-expressing neurons in the VNC (**a**). The scale bar is 100 μm. The reporter gene was not observed in the C57BL/6J mouse, in which the same viral vector was injected (**b**).

Supplementary Fig. 7

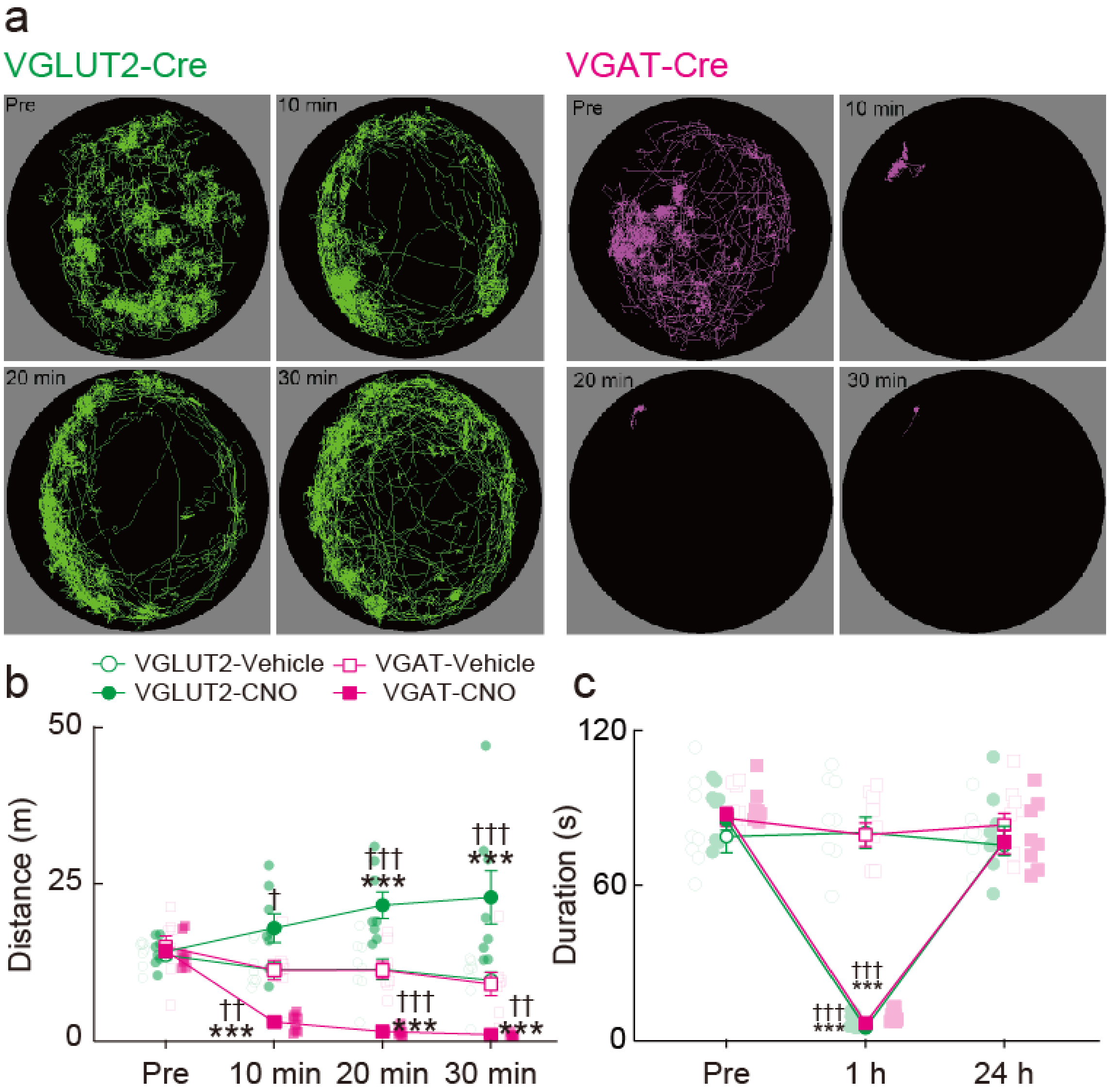

Supplementary Fig. 7: The effect of chronic activation of either the glutamatergic or GABAergic neurons in the vestibular nuclear complex (VNC) on the tracking movement

(**a**) The representative tracing of the movement before and after administration of clozapine N-oxide (CNO) in a VGLUT2-Cre and a VGAT-Cre mouse. The viral vector, AAV–CAG–FLEX–hm3D(Gq)–mCherry (AAV–hm3D(Gq)–mCherry) or its control vector, were injected in the VNC bilaterally in VGLUT2-Cre (n = 8) and VGAT-Cre (n = 8) mice. Each panel shows the total trace for 10 min. (**b**) Summarized data of the total distance of the movement for every 10 min in VGLUT2-Cre and VGAT-Cre mice. Two-way ANOVA with Tukey's multiple comparisons test; F (9,84) = 11.12, $P < 0.0001$ (Interaction), ***$P < 0.001$ vs. pre, †$P < 0.05$, ††$P < 0.01$, †††$P < 0.001$ vs. either VGLUT2-vehicle or VGAT-vehicle. (**c**) The duration of the rotarod experiment was measured before and after the administration of either clozapine N-oxide (CNO) or saline (vehicle). Each mouse underwent three sessions of the rotarod experiment, for which the average value was used. Data are means ± SEM (error bars). Two-way ANOVA with Tukey's multiple comparison tests; F (6,56) = 48.74, $P < 0.0001$ (Interaction), ***$P < 0.001$ vs. pre, †††$P < 0.001$ vs. either VGLUT2-vehicle or VGAT-vehicle. Detailed information on the statistical analyses is reported in Supplementary Table 2.

Supplementary Fig. 8

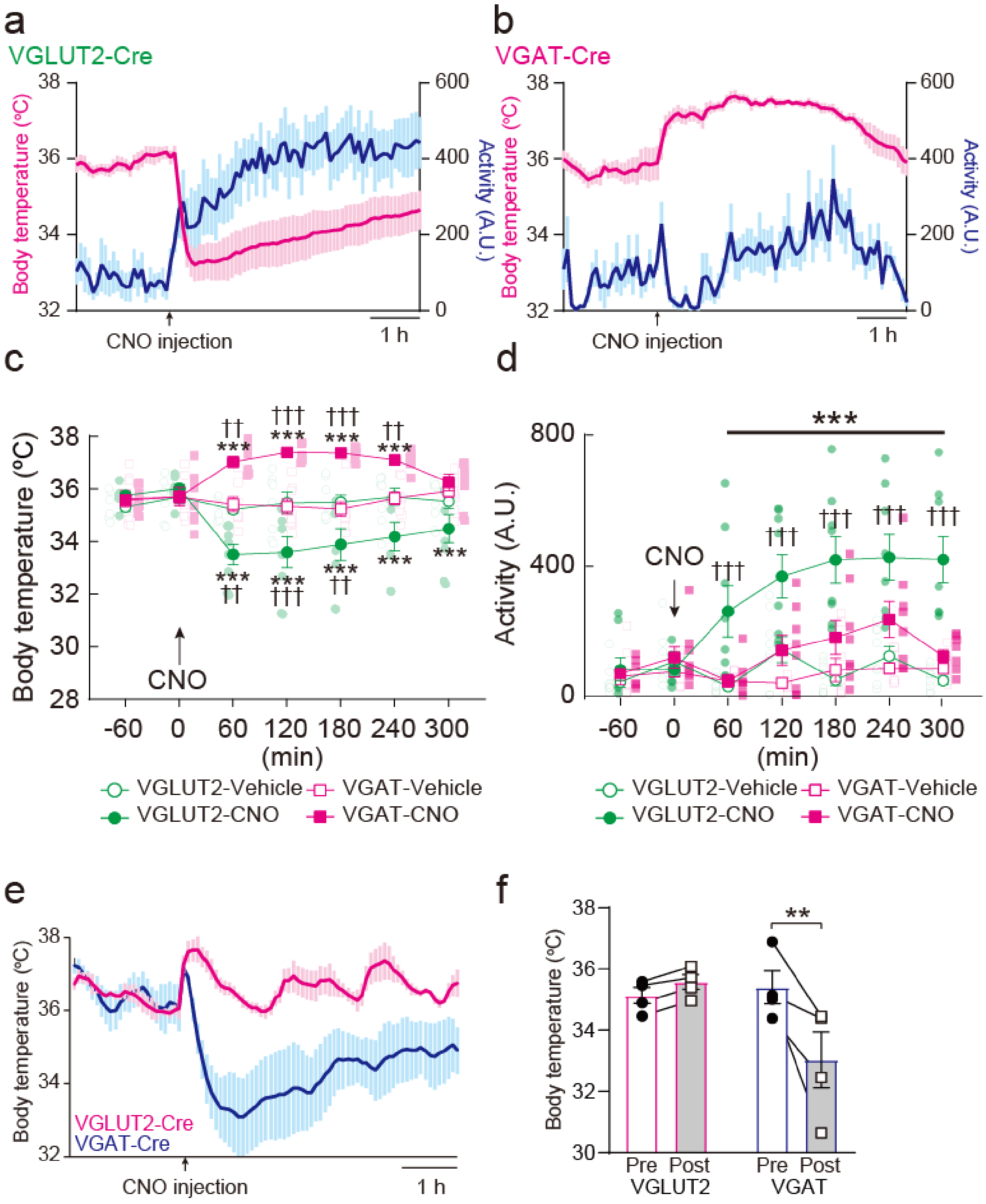

Supplementary Fig. 8: Changes in body temperature (BT) induced by unilateral chemogenetic stimulation of either glutamatergic or GABAergic neurons in the vestibular nuclear complex (VNC)

Averaged time-dependent changes in the BT (magenta line) and activity (blue line) induced by the administration of clozapine N-oxide (CNO) in the VGLUT2-Cre (**a**, n = 8) and VGAT-Cre (**b**, n = 8) mice. Either the viral vector, AAV–hm3D(Gq)–mCherry or its control vector, were injected in the VNC unilaterally. The arrow head shows the timing of CNO administration. The time scale bar represents 1 h. Data are means ± SEM (error bars). (**c**) Summarized data of the changes in the BT following the administration of CNO in the VGLUT2-Cre and VGAT-Cre mice. The arrow head shows the timing of administration of CNO. Each value was obtained by averaging the values every 60 min. For the statistical analyses, the values in -60 and 0 min were averaged as pre-values. Two-way ANOVA with Tukey's multiple comparisons test; F (15,140) = 9.210, $P < 0.0001$ (Interaction), *** $P < 0.001$ vs. pre CNO injection in either the VGLUT2-CNO or VGAT-CNO. ††$P < 0.01$ and †††$P < 0.001$ vs. either the VGLUT2-vehicle or VGAT-vehicle. (**d**) Summarized data of the changes in activity after the administration of CNO in the VGLUT2-Cre and VGAT-Cre mice. Each value was obtained by averaging the values every 60 min. For the statistical analyses, the values in -60 and 0 min were averaged as pre-values. Two-way ANOVA with Tukey's multiple comparisons test; F (15,140) = 5.516, $P < 0.0001$ (Interaction), ***$P < 0.001$ vs. pre CNO injection in VGLUT2-CNO; †††$P < 0.001$ vs. VGLUT2-vehicle. (**e**) Averaged time-dependent changes in the BT induced by the administration of clozapine N-oxide (CNO) in the VGLUT2-Cre (magenta line, n = 4) and VGAT-Cre (blue line, n = 4) mice. The viral vector, AAV2–DIO–hSyn–hm4D(Gi)–mCherry, was injected in the VNC unilaterally. The arrowhead shows the timing of CNO administration. The time scale bar represents 1 h. Data are means ± SEM (error bars). (**f**) Summarized data of the changes in the BT following the administration of CNO in the VGLUT2-Cre and VGAT-Cre mice. Two-way ANOVA with Tukey's multiple comparisons test; F (1,6) = 13.79, $P = 0.0099$ (Interaction). Double symbols indicate P < 0.01. Detailed information on the statistical analyses is reported in Supplementary Table 2.

Supplementary Fig. 9

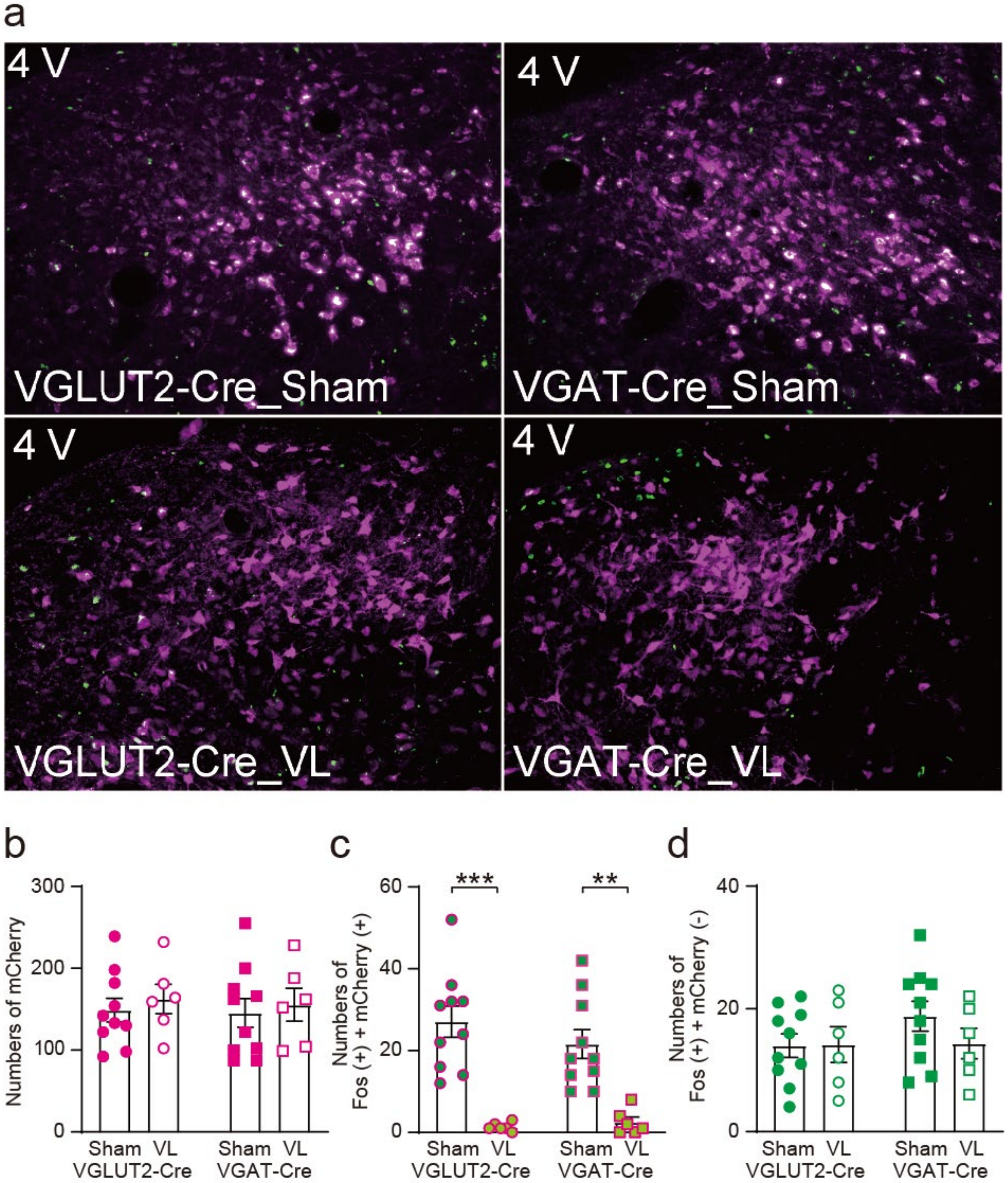

Supplementary Fig. 9: Expression of c-fos in either VGLUT2- or VGAT-expressing neurons located in the vestibular nuclear complex (VNC) induced by the 2g load.

(**a**) The viral vector, AAV–DIO–EF1α–mCherry, was injected in the VNC in the VGLUT2-Cre and VGAT-Cre mice. These mice were underwent vestibular lesion (VL) or its sham operation (Sham). The 2g load was applied for 90 min to examine the c-fos expression in the VNC. The scale bar is 100 μm, while 4 V represents the 4th ventricle. (**b-d**) Summarized data of c-fos expression in VGLUT2- and VGAT-expressing neurons in the VNC in mice with or without VL. Two-way ANOVA with Bonferroni's post-hoc test was applied. Double or triple significant symbols indicate $P < 0.01$, or $P < 0.001$, respectively. Detailed information on the statistical analyses is reported in Supplementary Table 2.

Supplementary Fig. 10

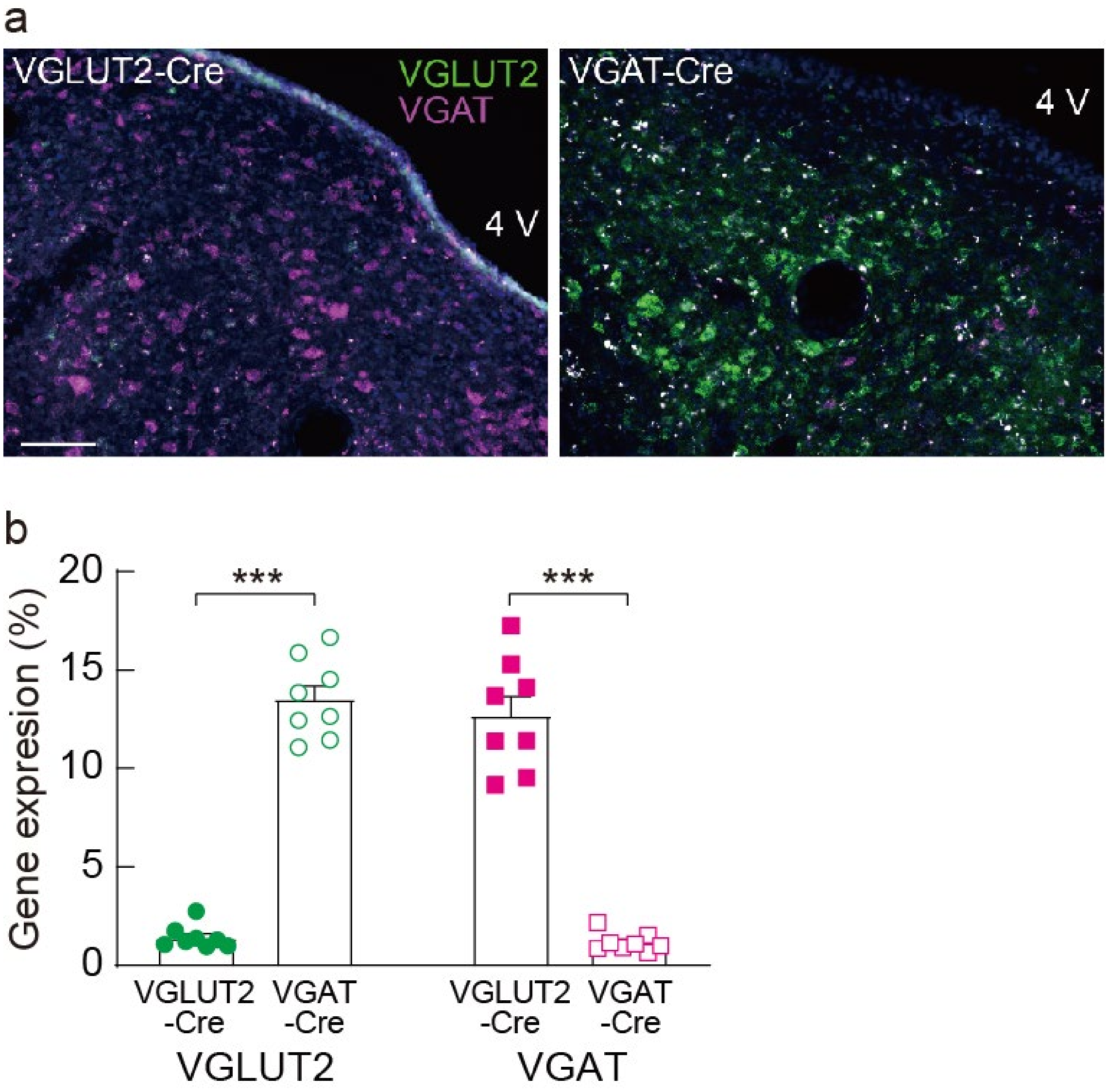

Supplementary Fig. 10

Deletion of VGLUT2 in the vestibular nuclear complex (VNC) using AAV2–DIO–taCasp3–TEVp

(**a**) The viral vector, AAV2- AAV2–DIO–taCasp3–TEVp, was injected in the bilateral VNC to delete VGLUT2-expressing neurons. For the Control group, the vehicle of the viral vector (Phosphate Buffered Saline) was used. The scale bar is 100 μm. (**b**) Summarized data of the VGLUT2 or VGAT expression using method of in-situ hybridization. The VGLUT2 or VGAT-positive area was normalized to total area of the region of interesting. A paired t-test was applied for the statistical analysis. Significant symbols indicate $P < 0.001$. Detailed information on the statistical analyses is reported in Supplementary Table 2.

Supplementary Fig. 11

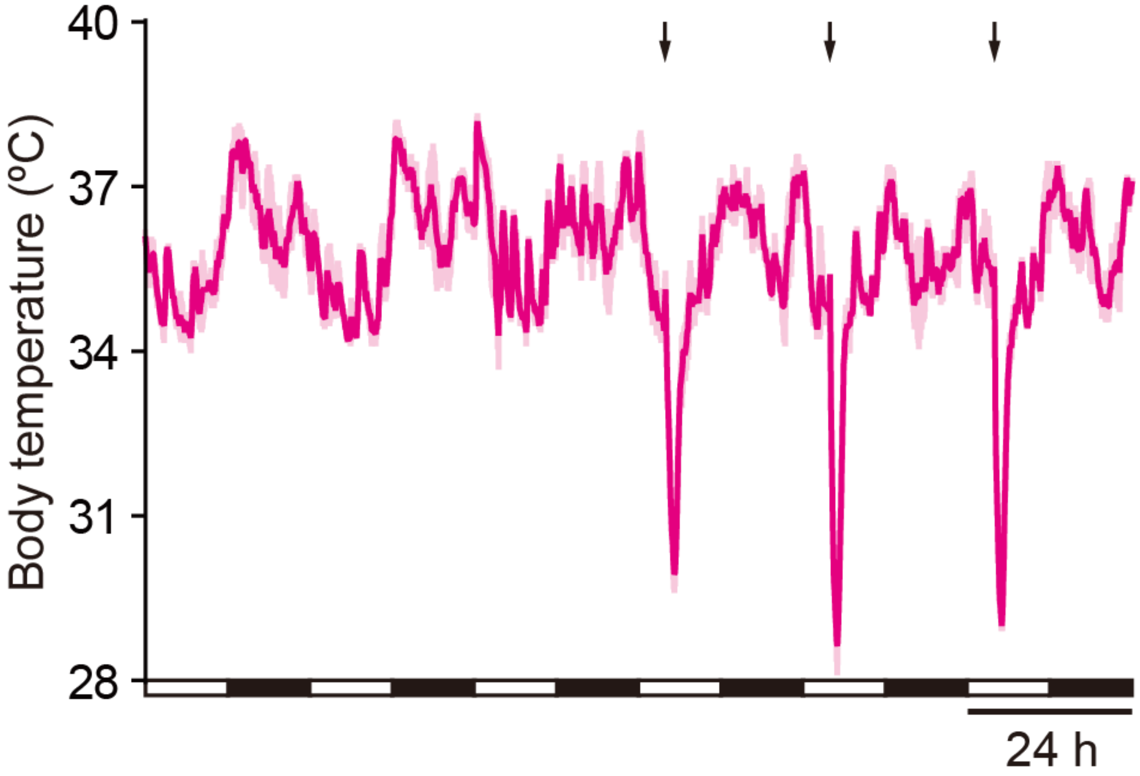

Supplementary Fig. 11: The effect of loading repetition on 2g-induced hypothermia
Averaged data of the changes in body temperature (BT) during repeated 2g load (n = 3). The triangular symbols indicate 2g load, which was applied from 11:00 to noon. Data are means ± SEM (error bars).

Supplementary Table 1

Information related to the drugs used for data illustrated in Figs. 1b and 1f

Name	Effect	Dose	Vehicle	Purchase
Hexamethonium	Ganglion blockade	50 mg/kg	Saline	Sigma
Isoprenaline	Nonselective beta adrenoreceptor agonist	10 mg/kg	Saline	Sigma
Adrenaline	Alpha and beta adrenoreceptor agonist	0.02 mg/kg	Saline	Daiichi-Sankyo
Yohimbine	Alpha 2 adrenoreceptor antagonist	2 mg/kg	Saline	Sigma
BRL-37344	Beta 3 adrenoreceptor agonist	0.5 mg/kg	DMSO	Sigma

Supplementary Table 2

Detailed information regarding the statistical analyses

Fig number	Test used	n	P value	F/t/z/R/ETC value
1c	Two-way ANOVA	8	< 0.0001 (Interaction)	F (7,98) = 25.19 (Interaction)
1d	Two-way ANOVA	8	< 0.0001 (Interaction)	F (7,98) = 4.896 (Interaction)
1e	Two-way ANOVA	7	< 0.0001 (Interaction)	F (1,12) = 66.81 (Interaction)
1g	Two-way ANOVA	4	< 0.0001 (Interaction)	F (3,12) = 77.18 (Interaction)
2b	Two-way ANOVA	8	< 0.0001 (Interaction)	F (1,14) = 166.4 (Interaction)
2c	Two-way ANOVA	8	< 0.0001 (Interaction)	F (4,56) = 59.31 (Interaction)
2d (NAd)	Two-way ANOVA	8	0.0477 (Interaction)	F (1,14) = 4.708 (Interaction)
2d (Ad)	Two-way ANOVA	8	0.1773 (Interaction)<br>0.0489 (Gravity)	F (1,14) = 2.018 (Interaction)<br>F (1,14) = 4.651 (Gravity)
2e (Sal vs. Iso, Ad, and Yoh)	One-way ANOVA	8	< 0.0001	F (3,28) = 17.53
2e (DM vs. BRL)	Unpaired t-test (two-tailed)	8	0.0278	t = 2.454, df = 14
3c	One-way ANOVA	8	< 0.0001	F (3,28) = 76.76
3d	One-way ANOVA	8	< 0.0001	F (3,28) = 72.99
3e	Two-way ANOVA	8	< 0.0001 (Interaction)	F (12,140) = 14.43 (Interaction)
3f	Two-way ANOVA	8	< 0.0001 (Interaction)	F (12,140) = 13.46 (Interaction)

4c	Two-way ANOVA	8	< 0.0001 (Interaction)	F (9,84) = 11.69 (Interaction)
4d	Two-way ANOVA	8	< 0.0001 (Interaction)	F (6,56) = 44.13 (Interaction)
5c	Two-way ANOVA	8	< 0.0001 (Interaction)	F (15,140) = 23.51 (Interaction)
5d	Two-way ANOVA	8	< 0.0001 (Interaction)	F (15,140) = 13.35 (Interaction)
6c	Two-way ANOVA	8	< 0.0001 (Interaction)	F (15,140) = 11.78 (Interaction)
6d	Two-way ANOVA	8	0.3311 (Interaction)<br>< 0.0001 (Time)<br>< 0.0001 (Groups)	F (15,140) = 1.135 (Interaction)<br>F (5,140) = 7.568 (Time)<br>F (3,28) = 14.98 (Groups)
6e	Two-way ANOVA	8	0.7692 (Interaction)<br>0.3040 (Time)<br>0.6392 (Groups)	F (6,56) = 0.5483 (Interaction)<br>F (2,56) = 1.216 (Time)<br>F (3,28) = 0.5704 (Groups)
7a	One-way ANOVA	8	< 0.0001	F (11,352) = 118.6
7c	Two-way ANOVA	8	< 0.0001 (Interaction)	F (15,140) = 14.32 (Interaction)
7d	Two-way ANOVA	8	0.0026 (Interaction)	F (15,140) = 2.507 (Interaction)
7f	Paired t-test (two-tailed)	8	0.0014	t = 5.081, df = 7
S2a	Two-way ANOVA	4	0.8415 (Interaction)<br>0.0581 (Duration)<br>0.0085 (Gravity)	F (3,12) = 0.2762 (Interaction)<br>F (3,12) = 3.290 (Duration)<br>F (1,12) = 9.853 (Gravity)
S2b	Two-way ANOVA	4	< 0.0001 (Interaction)	F (3,12) = 22.43 (Interaction)
S2c	Two-way ANOVA	4	< 0.0001 (Interaction)	F (3,12) = 18.23 (Interaction)
S4c	Two-way ANOVA	8	< 0.0001 (Interaction)	F (2, 42) = 13.05

S7b	Two-way ANOVA	8	< 0.0001 (Interaction)	F (9,84) = 11.12 (Interaction)
S7c	Two-way ANOVA	8	< 0.0001 (Interaction)	F (6,56) = 48.74 (Interaction)
S8c	Two-way ANOVA	8	< 0.0001 (Interaction)	F (15,140) = 9.210 (Interaction)
S8d	Two-way ANOVA	8	< 0.0001 (Interaction)	F (15,140) = 5.516 (Interaction)
S8f	Two-way ANOVA	4	0.0099 (Interaction)	F (1, 6) = 13.79
S9b	Two-way ANOVA	6 or 10	0.9213 (Interaction)	F (1, 28) = 0.009940
S9c	Two-way ANOVA	6 or 10	< 0.0001 (Group)	F (1, 28) = 42.58
S9d	Two-way ANOVA	6 or 10	0.364 (Interaction)	F (1, 28) = 0.8514
S10b	Unpaired t-test (two-tailed)	8<br>8	< 0.0001 (VGLUT2)<br>< 0.0001 (VGAT)	t = 16.29, df = 14<br>t = 11.36, df = 14
