# Supplementary material for: VGLUT2-expressing neurons in the vestibular nuclear complex mediate gravitational stress-induced hypothermia in mice
Source: Commun Biol. 2020 May 8;3:227. doi: 10.1038/s42003-020-0950-0 (PMC7210111; doi:10.1038/s42003-020-0950-0)
Supplement: Supplementary file 2 — Description of Additional Supplementary Files [file 42003_2020_950_MOESM2_ESM.pdf]

**Supplementary Movie 1: Photostimulation of either VGLUT2- or VGAT-expressing neurons located in the vestibular nuclear complex (VNC)**

Either channelrhodopsin-2 (ChR2) or archaerhodopsin (eArch) was expressed in VGLUT2- and VGAT-expressing neurons located in the left VNC. Photostimulation of each neuron induced either excitation through the ChR2 or inhibition through the eArch. The duration of photostimulation was 1 s.

**Supplementary Movie 2: Changes in mice behavior following the unilateral activation of VGLUT2-expressing neurons located in the vestibular nuclear complex (VNC)**

The viral vector, AAV–CAG–FLEX–hm3D(Gq)–mCherry (AAV–hm3D(Gq)–mCherry), was injected in the left VNC in a VGLUT2-Cre mouse. Clozapine N-oxide (CNO) was administered to activate VGLUT2-expressing neurons located in the VNC. The Movie is played at 10× speed.

**Supplementary Movie 3: Changes in mice behavior following unilateral activation of VGAT-expressing neurons located in the vestibular nuclear complex (VNC)**

The viral vector, AAV–CAG–FLEX–hm3D(Gq)–mCherry (AAV–hm3D(Gq)–mCherry), was injected in the left VNC in a VGAT-Cre mouse. Clozapine N-oxide (CNO) was administered to activate VGAT-expressing neurons located in the VNC. The Movie is played at 10× speed.

**Supplementary Movie 4: Comparison between the mice behavior induced by unilateral activation of VGLUT2- and VGAT-expressing neurons located in the vestibular nuclear complex (VNC)**

The viral vector, AAV–CAG–FLEX–hm3D(Gq)–mCherry (AAV–hm3D(Gq)–mCherry), was injected in the left VNC in either a VGLUT2-Cre or a VGAT-Cre mouse. The movie was recorded 60 min following clozapine N-oxide (CNO) administration. Note that tottering with body tilt to the right side (contralateral side to the activation) was observed in a VGAT-Cre mouse.

**Supplementary Movie 5: Changes in mice behavior following bilateral activation of VGLUT2-expressing neurons located in the vestibular nuclear complex (VNC)**

The viral vector, AAV–CAG–FLEX–hm3D(Gq)–mCherry (AAV–hm3D(Gq)–mCherry), was injected in the bilateral VNC in a VGLUT2-Cre mouse. Successively, clozapine Noxide (CNO) was administered to activate VGLUT2-expressing neurons located in the VNC. The Movie is played at 10× speed.

**Supplementary Data 1**

Source data underlying the graphs and charts presented in the main figures.
