# Supplementary material for: VGLUT2-expressing neurons in the vestibular nuclear complex mediate gravitational stress-induced hypothermia in mice
Source: Commun Biol. 2020 May 8;3:227. doi: 10.1038/s42003-020-0950-0 (PMC7210111; doi:10.1038/s42003-020-0950-0)
Supplement: Supplementary file 8 — Reporting Summary [file 42003_2020_950_MOESM8_ESM.pdf]

nature research

Corresponding author(s): Chikara Abe

Last updated by author(s): Apr 6, 2020

Reporting Summary

Nature Research wishes to improve the reproducibility of the work that we publish. This form provides structure for consistency and transparency in reporting. For further information on Nature Research policies, see Authors & Referees and the Editorial Policy Checklist.

Statistics

For all statistical analyses, confirm that the following items are present in the figure legend, table legend, main text, or Methods section.

n/a	Confirmed	
☐	☒	The exact sample size (*n*) for each experimental group/condition, given as a discrete number and unit of measurement
☐	☒	A statement on whether measurements were taken from distinct samples or whether the same sample was measured repeatedly
☐	☒	The statistical test(s) used AND whether they are one- or two-sided<br>*Only common tests should be described solely by name; describe more complex techniques in the Methods section.*
☐	☒	A description of all covariates tested
☐	☒	A description of any assumptions or corrections, such as tests of normality and adjustment for multiple comparisons
☐	☒	A full description of the statistical parameters including central tendency (e.g. means) or other basic estimates (e.g. regression coefficient) AND variation (e.g. standard deviation) or associated estimates of uncertainty (e.g. confidence intervals)
☐	☒	For null hypothesis testing, the test statistic (e.g. *F*, *t*, *r*) with confidence intervals, effect sizes, degrees of freedom and *P* value noted<br>*Give P values as exact values whenever suitable.*
☒	☐	For Bayesian analysis, information on the choice of priors and Markov chain Monte Carlo settings
☒	☐	For hierarchical and complex designs, identification of the appropriate level for tests and full reporting of outcomes
☒	☐	Estimates of effect sizes (e.g. Cohen's *d*, Pearson's *r*), indicating how they were calculated

Our web collection on statistics for biologists contains articles on many of the points above.

Software and code

Policy information about availability of computer code

Data collection	*Provide a description of all commercial, open source and custom code used to collect the data in this study, specifying the version used OR state that no software was used.*
Data analysis	GraphPad Prism 8

For manuscripts utilizing custom algorithms or software that are central to the research but not yet described in published literature, software must be made available to editors/reviewers. We strongly encourage code deposition in a community repository (e.g. GitHub). See the Nature Research guidelines for submitting code & software for further information.

Data

Policy information about availability of data

All manuscripts must include a data availability statement. This statement should provide the following information, where applicable:

- Accession codes, unique identifiers, or web links for publicly available datasets
- A list of figures that have associated raw data
- A description of any restrictions on data availability

The data that support the findings of this study are available from the corresponding author upon reasonable request.

Field-specific reporting

Please select the one below that is the best fit for your research. If you are not sure, read the appropriate sections before making your selection.

☒ Life sciences ☐ Behavioural & social sciences ☐ Ecological, evolutionary & environmental sciences

Life sciences study design

All studies must disclose on these points even when the disclosure is negative.

Sample size

We determined that the body temperature before 2g loading is 34.91, the body temperature during 2g loading is 30.45, standard deviation is 2.45 (sd), maximum number of pairwise comparisons is 6 (tau), type I error is 0.05 (alpha), and power is 0.8 (power). We measured sample size using R as follows.

```
mA=34.91
mB=30.45
sd=2.45
tau=6
alpha=0.05
power=0.8
(n=2*(sd*(qnorm(1-alpha/(2*tau))+qnorm(power))/(mA-mB))^2)
ceiling(n)
```

This examination shows that the sample size is 8, so we used 8 mice for each group in this study.

Data exclusions

No data were excluded from the study. However, there were some non-responders (no complete vestibular lesion (VL), no body tilt by photostimulation, and no changing in behavior by the chemostimulation) in the experiment. These mice were excluded before data acquisition.

Excluded mice
VL mice, n = 4: They could swim (Yamaoka et al., J Neurosci Methods, 2017).
Optogenetics mice, n = 3: They did not show the body tilt by the photostimulation.
Chemogenetics mice, n = 1: A mouse did not show the changing in behavior.

Replication

The same experiment, measurement of drop in body temperature, was conducted by three researchers (CA, YY, and HM). Similar response was observed thus we confirmed the experimental replication.

Randomization

All mice were randomly picked up for the measurement in the study. This was applied including "male and female", "viral vector and its control" and "drugs and their control vehicle".

Blinding

Although the surgery and the measurement were conducted by the same person (CA), the cage tag which has a group name was masked by the tape. Furthermore, all raw data were always checked by 2 to 3 researchers together (CA, YY, and HM).

Reporting for specific materials, systems and methods

We require information from authors about some types of materials, experimental systems and methods used in many studies. Here, indicate whether each material, system or method listed is relevant to your study. If you are not sure if a list item applies to your research, read the appropriate section before selecting a response.

Materials & experimental systems

n/a	Involved in the study	
☐	☒	Antibodies
☒	☐	Eukaryotic cell lines
☒	☐	Palaeontology
☐	☒	Animals and other organisms
☒	☐	Human research participants
☒	☐	Clinical data

Methods

n/a	Involved in the study	
☒	☐	ChIP-seq
☒	☐	Flow cytometry
☒	☐	MRI-based neuroimaging

Antibodies

Antibodies used

anti-DsRed (rabbit polyclonal, 1:500; Clontech #632496; Clontech Laboratories)
anti-GFP (chicken polyclonal, 1:500; GFP-1010; Aves Labs)
anti-c-fos (1:1,000; Millipore #ABE457; EMD Millipore)
Alexa Fluor-488-tagged rabbit anti-chicken antibody (1:200; Jackson ImmunoResearch Laboratories)
Alexa Fluor-594-tagged donkey anti-rabbit antibody (1:200; Jackson ImmunoResearch Laboratories)
Alexa Fluor-488-tagged donkey anti-rabbit antibody (1:200; Jackson ImmunoResearch Laboratories).

Validation

Antibodies except for anti-c-fos were used in the previous study (Abe et al., Nat Neurosci, 2017). The validation of the antibody for anti-c-fos has been already confimed (http://www.merckmillipore.com/JP/ja/product/Anti-c-Fos-Antibody,MM_NF-ABE457).

Animals and other organisms

Policy information about studies involving animals; ARRIVE guidelines recommended for reporting animal research

Laboratory animals	C57BL/6J mice, male and female, 8-12 weeks<br>VGLUT2–Cre (STOCK Slc17a6tm2(cre)Lowl/J) mice, male and female, 8-12 weeks<br>VGAT–Cre (STOCK Slc32a1tm2(cre)Lowl/J) mice, male and female, 8-12 weeks
Wild animals	The study did not involve wild animals.
Field-collected samples	The study did not involve samples collected from the field.
Ethics oversight	The animals used in the present study were maintained in accordance with the "Guiding Principles for Care and Use of Animals in the Field of Physiological Science", set by the Physiological Society of Japan. The experiments were approved by the Animal Research Committees of Gifu University.

Note that full information on the approval of the study protocol must also be provided in the manuscript.
